# Five-Dimensional Positional Modulation with Quench-Trapped Modulation Phase in Solid-state Electrolyte Network Solid

**DOI:** 10.1063/4.0000954

**Published:** 2025-10-27

**Authors:** M. Brody Mistrot, Michael J. Zdilla

**Affiliations:** Temple University, Philadelphia, PA, USA

## Abstract

Here we report on an interpenetrated network solid:(Adpn)3Zn(BF4)2 which exhibits multiple crystallographically modulated phases; one of which is only accessible by quench-trapping. This material was originally intended as a solid-state electrolyte for zinc batteries, but during structural characterization satellite peaks were observed in the diffraction pattern, indicating higher dimensionality of the lattice. After initial datasets at 100K proved difficult to analyze, full spheres were collected at 298K, 250K, 200K, and 150K (Figure 1). This series showed transitions between two modulated phases: one between 250K and 200K, dubbed the ‘simple modulation,’ characterized by satellites at 1/3,1/3,0 and another pattern which only appears when the sample is quench-cooled to 100K. Above 250K, the reciprocal lattice shows no satellite peaks, and the lattice loses long-range positional periodicity, becoming disordered (Figure 2). Upon investigation of the moderate temperature modulation, the data was first treated as a (3+1)D incommensurate modulation, but this is not the correct description, and the refinement proved unstable. Upon further review, the correct description of this system is instead a (3+2)D commensurate modulation with a symmetry equivalent q2 vector required to deconvolute the overlapping satellite intensities from neighboring main peaks. This description has proved difficult to treat explicitly using modulated structure refinement in JANA, and only an average structure (subcell) and two brute-forced super-cell structures have been solved using the ShelX suite (Figure 3). When quench-cooled to 100K, this system exhibits an unusual phenomenon: quench-trapped modulation. When cooled gradually under a cryo-stream from room temperature the pattern of satellite peaks does not change between 150K and 100K. However, if the crystal is quenched from room temperature, a second set of satellites at 1/6,1/6,0 appear alongside the previously described 1/3,1/3,0 satellites. We hypothesize that these additional satellites can be attributed to a second modulated phase which becomes kinetically trapped when quenched. If allowed to warm, even slightly, this phase relaxes down to the 1/3,1/3,0 phase and becomes inaccessible unless the sample is thawed and re- quenched. While technically solvable in JANA, this data would need to be treated as a twin of two distinct modulated phases. Such a solution would require immense effort to produce and would be unlikely to elucidate any new information not already obtained by the disordered, average, or super-cell structures of this system. Despite the difficulty in determining a structure, the quench vs slow-cool effect is clear. The complexity and difficulty found in these data reinforce higher-dimensional structures as a forefront in crystallography which require further methods development.

